# Adolescent paediatric transplant patients’ experiences of transition to adult services

**DOI:** 10.4102/hsag.v27i0.1937

**Published:** 2022-10-27

**Authors:** Lesley Prentice-Hoogervorst, Pat M. Mayers

**Affiliations:** 1 Division of Nursing and Midwifery, Faculty of Health Sciences, University of Cape Town, Cape Town, South Africa

**Keywords:** adherence, adolescent, chronic illness, non-compliance, renal or liver transplant, transition

## Abstract

**Background:**

Transition of adolescents from paediatric to adult health services is an important aspect of caring for young people with chronic diseases. Successful transition of adolescents living with a transplant is critical for long-term survival into adulthood. This qualitative study explored the lived experience of adolescents in a South African setting following their planned and supported transition process.

**Aim:**

To explore the lived experience of transition for adolescents who had received a renal or liver transplant as a child, from paediatric to adult transplant services in the public health sector.

**Setting:**

Cape Town, South Africa.

**Method:**

Six purposively sampled adolescents, who had participated in a planned transition from a tertiary level children’s hospital to the affiliated adult hospital, were interviewed in the setting and language of their choice. The transcribed interviews were analysed using an interpretative phenomenological analysis (IPA) approach.

**Results:**

Five themes emerged: living with uncertainty in a changing world; being known, not knowing, and knowing; ambivalent relationships; the journey of loss; and being heard. Pre-transition planning, support groups, and consistency of clinicians within an accepting environment facilitated the adolescents’ individuation and acceptance of self-care responsibility.

**Conclusion:**

Carefully planned, collaborative preparation and implementation of a facilitated psycho-supportive intervention for transition can facilitate adolescent cooperation and adherence, minimise the risk of psychological and medical sequelae, and support the adolescent in adapting to living with a transplant as an adult.

**Contribution:**

This study offers insight into the importance of planned and supported transitional care of adolescents living with an organ transplant.

## Background

The management of chronic and progressively deteriorating illnesses in children has improved significantly with increasing numbers of children reaching young adulthood (Fazel et al. 2021; Murphy Salem & Graham 2021). Extended survival has meant that the transition of the chronically ill adolescent from paediatric to adult care is a necessary, yet complex phenomenon (Francis et al. 2018). As adolescence is a time of developmental transition, clinicians need to be aware of normal developmental issues, to understand the impact a chronic condition may have on the child’s behaviour during adolescence and the transition from paediatric to adult health services (Fazel et al. 2021). The life stage of adolescence is increasingly recognised as a specialist area of medical practice (Lee et al. 2016).

### Normal adolescent development

Adolescence is a preparatory transitional stage between childhood and adulthood, during which a sense of personal identity is formed (Erikson & Rapaport 1959). Cultural and socio-economic circumstances, previous life experiences, peer group, and family relationships influence how the child deals with the resurgence of childhood anxieties and the bio-psychosocial changes of rapid physical growth, sexual maturation, and transformation in thinking patterns of adolescence (eds. Martin, Bloch & Volkmar 2018; Viner, Allen & Patton 2017). In low-middle income countries (LMICs) such as South Africa, adolescents are vulnerable because of stressors such as substance abuse, conflict and breakdown of the family unit, exposure to trauma, scholastic delays, and financial pressures (Hall et al. 2019; Killian et al. 2018).

### The effects of chronic illness and hospitalisation on children and adolescents

A diagnosis of life threatening, and acute or chronic illness is an unexpected trauma which frequently requires admission of children in an acute or critical condition, into an unfamiliar hospital environment (Marsac et al. 2014). Children with chronic renal or liver disease require the life-saving intervention of organ transplantation, which offers increased length and quality of life but requires life-long medical care (Steuer & Opiola McCauley 2017). New social and emotional roles are explored during normal adolescent development. These are complicated in the presence of chronic and/or life-threatening illness by unpredictable relapses and hospitalisations (Maor & Mitchem 2020). Physically, the illness process and medication may result in stunted growth and distorted features (Watson et al. 2018). Factors such as an increased awareness of body image, need for peer approval, and rapid physical and emotional changes compound pressures faced by chronically ill adolescents, which may result in school absenteeism, poor academic progress (Lum et al. 2017), and maladaptive psychological and physical functioning (Lozano & Houtrow 2018).

### Adolescent transition

Transitional care is defined by the Society for Adolescent Medicine as the ‘purposeful, planned movement of adolescents and young adults with chronic physical and medical conditions from child-centred to adult-oriented health-care systems’ (Blum et al. 1993:570). The goal is to maximise lifelong functioning and potential through the provision of high-quality, developmentally appropriate, uninterrupted healthcare services (National Institute for Health and Care Excellence 2016), which is a challenge for the healthcare team (Crawford et al. 2020). Effective intervention planning in transitional care is imperative to improve the adolescents’ quality of life as life expectancy improves (Francis et al. 2018).

Transition from paediatric to adult care coincides with the normal developmental phase of early adolescence (age 10–13 years), with the onset of explorative behaviour patterns and the normal, but unpredictable developmental and physiological changes (National Academies of Sciences, Engineering, and Medicine 2019). Chronological age, however, does not always match with maturation and developmental age (Dallimore, Neukirchinger & Noyes 2018). Uncertainties generated during adolescence and the unfamiliarity of the adult unit and staff post-transfer may result in the adolescents becoming non-compliant with their previously regular treatment regimens (Killian et al. 2018; Richards et al. 2018). Dallimore et al. (2018) highlighted that transition of adolescents with transplants is ‘a zone of conflict between independence and dependency with young people feeling powerless on one hand and overwhelmed on the other’ (p. 1). Interventions to reduce morbidity and mortality of young adult and adolescent kidney transplant patients include pre-transplant evaluation, transition support, medication adherence strategies, and effective monitoring (Richards et al. 2018).

Specialised public health paediatric services in South Africa treat children up to the age of 13 years, after which they are expected to transition to adult care (Shung-King et al. 2019). South Africa currently has no national policy regarding the transition of care and has few transitional care guidelines.

### Non-adherence in adolescent transition

Non-adherence is a life-threatening behaviour which may lead to a relapse of the chronic condition, graft loss in patients with organ transplants, or at worst, death (Tang et al. 2021). The transition in medical management from paediatric to adult care during adolescence involves change and the loss of familiar longstanding attachment relationships in the paediatric setting between families, patients, and the clinical staff who have been involved in the child’s care (Francis et al. 2018). Anxieties originating from earlier life experiences may re-emerge, leading to negative coping mechanisms or defensive behaviours (Colonnesi et al. 2011) with a resulting deterioration of the adolescent’s adherence and health status (Shemesh et al. 2018). Renal transplant recipients aged 14–16 years have the greatest risk of kidney allograft failure (Andreoni et al. 2013). McCulloch et al. (2006) in a review of the paediatric transplant programme in the study setting, reported that five stable adolescent renal transplant patients lost their grafts within 1 year of transfer, two of whom died. High rates of non-compliance, rejection, and graft loss remain a concern in South Africa (Chhiba et al. 2022). Transition in the study setting has often been postponed until later adolescence and a consultation liaison team was established to provide transition support, of which the first author, a psychiatric nurse specialist, was a member. Limited research regarding the adolescent transition to adult care has been published in South Africa (Chhiba et al. 2022). This study thus aimed to explore the lived experience of adolescents following their transition.

## Conceptual framework

This study has drawn on the work of Bowlby and Ainsworth in relation to attachment theory in infancy (Ainsworth et al. 2015; Bowlby 2005). A secure attachment relationship in the first 18 months of life positively affects ‘the internal working model’ of the child (Calam 2001:3) which, in turn, influences the child’s ability to form new relationships and adapt to change (eds. Grossmann, Grossmann & Waters 2005). Attachment theory explains the child’s need to develop a secure dependence on the parent before moving into unfamiliar situations, which echoes the developmental task of adolescent individuation from the parent (Calam 2001; eds. Grossmann et al. 2005). The normal function of the attachment figure during childhood growth and development is to protect and comfort the child (Colonnesi et al. 2011). In the event of a life-threatening illness, such as acute renal failure, the parent is unable to provide sufficient care. Instead, the child experiences hospitalisation, life-saving procedures, and ultimately a transplant, which would force the child to acquire ways of self-soothing (Bowlby 2005).

## Design and methods

A qualitative research design, using interpretative phenomenological analysis (IPA), was used in the study. Phenomenology, the psychological study of subjective experience, was appropriate for exploring the lived experience of transition for adolescents who had received an organ transplant (Peat, Rodriguez & Smith 2019). Interpretative phenomenological analysis aims to provide ‘detailed examinations of personal lived experience’ (Smith & Osborn 2015:41) and meanings attributed to particular experiences (Smith, Flowers & Larkin 2009), and is situated in the intuitive, empathic paradigm (Pietkiewicz & Smith 2014).

### Study population and setting

The study was conducted at the adolescent clinic of an adult tertiary level hospital in the Western Cape, South Africa. We purposively selected six patients in late adolescence (18–22 years of age), two males and four females, who had received a transplant as a child; two participants had received liver transplants and four had received a kidney transplant. All participants had transitioned from paediatric to adult services in the 6 months prior to the study. Transition had been delayed because of concern about graft loss if transitioned in early adolescence. All participants had been exposed to pre-transplant workshops and were attending the adult renal or liver clinics at the adult tertiary hospital. Mediated access was obtained with the assistance of the consulting physicians. Patients who met the inclusion criteria were given information about the study and voluntary informed consent was obtained.

### Ethical considerations

Ethics approval was obtained from the University of Cape Town Health Research Ethics Committee (HREC Ref. 171/2010) and permission granted by the facility review board of the study setting, the study conformed to the Declaration of Helsinki (World Medical Association 2008). Confidentiality, anonymity, and protection of vulnerable participants were assured. Withdrawal from the study at any time without penalty or withdrawal of therapeutic services was guaranteed. Counselling support was available should a participant experienced distress during or after the interview (Dempsey et al. 2016; Houghton et al. 2010). There were no direct benefits for the participants; however, the opportunity to tell a story may have benefits (Nurser et al. 2018).

### Data collection

The first author conducted in-depth interviews with each participant in a private setting of the participant’s choice. The IPA interview facilitates the sharing of personal information, thoughts, and feelings (Engward & Goldspink 2020; Smith et al. 2009). Interviews of approximately 60 min duration were audio-recorded; five in English and one in Afrikaans which was later transcribed into English. A consistent interview approach was employed with all participants, who were encouraged to share their experiences in their own words, with minimal prompting. Recordings were transcribed verbatim and checked for accuracy by both authors. The translated transcript was validated by an independent Afrikaans editor.

### Data analysis

Interpretative phenomenological analysis is a cyclical process whereby the researcher proceeds through iterative stages: first encounter with the text; preliminary themes are then identified; themes are grouped together as clusters; themes are tabulated in a summary table (Peat et al. 2019; Smith et al. 2009). Each interview was individually analysed; the researchers read and reread the transcripts, bracketed any personal responses, and noted the meanings and significance of the individual’s experience emerging from the text. Field notes and journal entries were taken by the first researcher and discussed with the second. Reflexivity in qualitative research is affected by the researcher’s familiarity with the setting and study participants, and the researchers maintained an awareness of the impact of personal experiences and possible bias on the rigour and credibility of the research (Berger 2015; Engward & Goldspink 2020).

### Data management and trustworthiness

All recorded and transcribed data were archived in a password-protected computer. Confirmability and dependability were ensured through the inclusion of quotes from the transcribed interviews. A clear audit trail of activities, reflective discussions, and decisions was documented throughout the study.

### Findings

Five themes emerged which describe the adolescent’s lived experience of living with a transplant and transition to adult care (Table 1). The adolescent transitions not only from a paediatric to an adult healthcare setting, but through a personal journey in which he or she must deal with uncertainty, ambivalence, losses, and learning to live as a transplant recipient.

**TABLE 1:** Themes and sub-themes.

Theme	Sub-theme
Living with uncertainty in a changing world	*The struggle of being; an adolescent living with a transplant* *Adolescent transition to adult services* *The impact of medication*
Being known, not knowing, and knowing	*Being seen and being known* *Not knowing*
Ambivalent relationships	*Multiple ambivalences – mother; home; family; self; friends; the organ donor; the hospitals*
The journey of loss	*Loss of the healthy child* *Transplantation: Loss of dependence and care*
Being heard	*Spirituality and the power of belief*

### Living with uncertainty in a changing world

For each participant, becoming a patient with a chronic, life-threatening condition in early childhood was a life-changing experience. Accurate diagnosis required multiple investigations, stabilising treatment regimens and admissions, and eventually an organ transplant.

#### The struggle of being an adolescent with a transplant

Adolescent developmental tasks include self-identity, peer acceptance, and adjustment to physical and emotional changes. Participants became acutely aware of a changing body:

‘… I have a lot of cuts and have gone through so much, I do not like anyone to touch me. I feel like they may say “no, your cuts are ugly”, that is why I wear long sleeved T-shirts.’ (Participant 5, Female, age 20)

Post-operative management, critical to the success of the transplant, is a complex routine for the patient, health professionals, and the family, often necessitating separations because of hospitalisation: ‘Where I was staying … my mother was not here, she just visited holidays sometimes …’ (Participant 6, Female, age 18). Medication affected their physical appearance and in turn their sense of identity: ‘Look at her she has a big stomach. I think she’s pregnant’ (Participant 3, Female, age 19). Transplant regime restrictions inhibited the participants’ freedom to enjoy social activities, as they had to balance their desire for peer acceptance while protecting their health: ‘I was always feeling like the odd one out of a situation’ (Participant 3, Female, age 19).

#### The impact of medication

The complex medication regimen is an integral part of the recipient’s lived experience, controlling all aspects of daily living:

‘… It’s like … a part of my life, a routine. I have to get up in the morning, take my medication in the afternoon, in the evening … I travel with my medication. I don’t think – “ok I will be back before the time of the medication” … so wherever I am I know it’s that time … ok now it’s medication time, stop whatever you are doing … ’ (Participant 2, Male, age 20)

Medication side effects are difficult to cope with: ‘… prednisone … makes us eat a lot, and I used to get fatter and fatter … and after that I became depressed …’ (Participant 3, Female, age 19). Managing the demands of post-transplant care is difficult:

‘… With a transplant – it’s very hard because of medication and I get sick, must eat the medication all the time, … it’s not good at all … it was better when I was small but now, I’m growing up it’s very too hard.’ (Participant 6, Female, age 18)

For the participants, the uncertainty of living with a transplant meant facing their own mortality and that of fellow patients at a life stage where healthy adolescents give little thought to disability or death: ‘… every year I hear some friends that are gone … and this year it’s [*my friend*] … so … maybe I’m going to be like that ….’ (Participant 6, Female, age 18).

#### Adolescent transition to adult services

Despite the transplant-related restrictions, participants moved through the developmental stages of individuation toward self-acceptance within the safety and consistency of care at the children’s hospital. Transition from the familiarity of the children’s hospital to the unknown, unfamiliar adult service was experienced as rejection by their hospital ‘family’. They spoke of inadequate or no preparation prior to transition: ‘… during the year they told me like December will be your last appointment here then you will be transferred …’ (Participant 2, Male, age 20). ‘And [the doctor] said, well this would be your last day at [the children’s hospital]’ (Participant 4, Female, age 22).

Participants responded in varied ways. For this participant, it was seen as inevitable: ‘[*That*] is a children’s hospital, you not going to be there forever. That’s what you have to accept, you gonna have to move to the adult’s stage……’ (Participant 2, Male, age 20); demonstrating learned compliance of a chronically ill child ‘… so I told them, ok …’ (Participant 2, Male, age 20). Participant 4 expressed a sense of shock and loss, assuming that she was not a ‘good enough’ patient:

‘… I started to stutter because I … I couldn’t understand what I did wrong, I felt like I … I’m being punished … I started crying, and I said … I’m not leaving [this hospital] …. I want to stay here. I’m not ready …’ (Participant 4, Female, age 22)

### Being known, not knowing, and knowing

The children’s hospital with its familiarity, predictability, and consistency of routine provided safety and support. Being known and accepted, despite their illnesses and limitations, helped participants cope with uncertainty, fear, and repeated hospitalisation. Transition meant a loss of this sense of familiarity: ‘It was the most difficult thing ‘cos in [the children’s hospital] … you used to know … everyone who passed by. And they knew us’ (Participant 1, Male, age 20). The fear related to the uncertainty (not knowing) at the onset of the illness journey was mitigated by the trust and security developed over the years of regular contact with the children’s hospital. The move to the adult hospital reawakened the insecurity, loss of identity, and anxiety, and the unknown adult hospital was experienced as frightening and different:

‘… It’s like preparing you for your death sentence the first time you enter this hospital, (voice softer, lower) but this is what creeps me out, just the bigness and it’s cold and everything seems like a jail … oh gosh it’s really huge inside.’ (Participant 4, Female, age 22)

Their resistance to the change engendered feelings of abandonment and avoidance: *‘…* like … not wanting to come to the hospital anymore, skipping a dose … even staying away. Well, eventually you would become ill, and you’d be rushed into the hospital …’ (Participant 2, Male, age 20).

The preparatory workshops and adolescent groups facilitated clarity and a sense of knowing about the move and reduced anxiety: *‘*What helped me … we were welcome there and those sessions that they used to have at [the children’s hospital] that helped us a lot …’ (Participant 1, Male, age 20). The presence of familiar paediatric physicians at the adult facility helped to alleviate anxiety and fears ‘Cos this person is the Doc, he’s not just doing his job he’s showing caring … and that like really comforts me’ (Participant 2, Male, age 20).

### Ambivalent relationships

Ambivalent relationships permeated every area of the participants’ lives. People who were seen as supportive could as easily be harsh: ‘Sometimes I did forget to take medication so … (Laugh) then my Mommy would shout at me or make me or tell me …’ (Participant 3, Female, age 19); ‘mothering’ was given by the primary caregiver, but also by the staff in the hospital: ‘ … the comfort of the nurses and doctors. Whenever I wanted something, I used to get it’ (Participant 1, Male, age 20). Home was a place in which they were cared for but could not always be kept safe: ‘But I became so sick at home that I was admitted to hospital on my death bed’ (Participant 5, Female, age 20). The attachment relationships with their own mothers and the ‘hospital as mother’ became ambivalent, particularly in times of crisis when participants felt that the hospital was safer; a space associated with pain, but also with safety and healing: ‘In and out of hospital so that the machines could clean my abdomen via an artificial kidney and then sent home again to do this at home’ (Participant 5, Female, age 20).

The participants developed a sense of belonging over the years of chronic illness and being treated as special in the hospital environment. The hospital became a ‘home from home’ and staff felt like parents and siblings: ‘I call that family. That’s people I trust for all my years ever since I’ve been there’ (Participant 4, Female, age 22). For this participant, the hospital was: ‘the best (laughter) … the way the nurses treated you and most of the times I would want to be more at hospital than at home’ (Participant 3, Female, age 19).

Although the transplant decreased the frequency of illness and hospitalisation, there was a new responsibility of care for the transplanted organ. Medication was a non-negotiable part of treatment: ‘The doctors are so clever they can see in your blood if you have not taken your tablets, so I take them with me wherever I go’ (Participant 5, Female, age 20). Visible scars affected body image and awareness of still being different. It was difficult to be ‘normal’ as they were reminded about their limitations: ‘… my mom and them [*the hospital staff*] will remind me ‘hey you can’t do this, you know, you had a transplant, look at your stomach…’ (Participant 2, Male, age 20).

The interplay of individuation, power, and control played out in the narratives. A second chance at life, at the cost of loss of another life, left participants with a hope that the donor family might be comforted by the gift of life and health to another: (Participant 2, Male, age 20) ‘ *…* his family donated their loved one’s [organ] to you and they don’t wanna see it go to waste … I think that would really bring some comfort to them *…*’ and anxiety about the potential impact of the donor’s personality on their own, a need to find common ground with the anonymous donor ‘… so maybe there could be like something in common …. maybe he liked cars, I like cars … ’ (Participant 2, Male, age 20). There was for this participant a sense that she should be grateful for being given a chance to live when the donor had died *‘*I wouldn’t disappoint her and the rest of the doctors no matter what and the guy who gave [*the kidney*] to me even though we never communicated …’ (Participant 4, Female, age 22).

Relationships between self and others were complex and marked by ambivalence about committing to relationships and friendships or exposing their ‘real self’ based on previous negative experiences of rejection and/or humiliation because of their physical limitations. ‘I just have one friend who knows I have kidney problem’ (Participant 6, Female, age 18). Social activities with potentially negative health consequences were restricted, such as alcohol consumption and contact sports: ‘I can hang with you guys, but I’m not allowed to do this … my health is gonna be in danger … ’ (Participant 2, Male, age 20). The strict regime left little room for natural adolescent exploration and experimentation.

### The journey of loss

From the onset of the illness, participants began an unsafe and unpredictable journey. Health fluctuations affected their mood and ability to manage day-to-day activities and relationships. Participants spoke of feeling different: ‘Maybe if I didn’t have the transplant, things would have been different, I wouldn’t be here right now, or I wouldn’t be like where I am’ (Participant 3, Female, age 19). To cope with this, they often presented a façade of pseudo-maturity and coping as a defensive strategy to deal with their fears, the repeated loss of fellow patients, to live up to the adult caregivers’ perceived expectations. This façade disappeared when alone:

‘[*S*]ometimes when I’m in bed I’m always thinking … why I’m like this, the tears will just come. I’ll cry and my mother would not see me ‘cos she’ll be sleeping and I’m not telling her why.’ (Participant 6, Female, age 18)

#### Transplantation: Loss of dependence and care

Transplantation is the culmination of an extended and anxious period of waiting, sometimes up to five years, for a suitable organ. The excitement, anxiety, and sense of relief at being given a second chance at life were tempered by the lifelong post-transplant restrictions, ongoing treatment regime, the threat of organ rejection, and the burden of self-care after the transplant.

### Being Heard

The sense of belonging, experienced in the children’s hospital, was lost in the participants’ transition to the unfamiliar adult hospital. They felt they did not belong and were not accepted by the adult hospital treatment team.

In the psycho-supportive adolescent group, they felt heard in a safe, accepting space for the expression of these anxieties and fears. This provided participants with the opportunity to hear and share the struggles of peers in a similar position within a confidential space: ‘Because we are showing sharing each other how we are feeling and who are we. It is a good thing’ (Participant 6, Female, age 18). Participant 4 added: ‘… you know it’s just gonna stay in these four walls ….’ (Participant 4, Female, age 22)

#### Spirituality and the power of belief

It was often difficult to reconcile spirituality, beliefs, and the experience of illness. Responses to ‘being different’ varied. Participant 6 felt unheard, uncared for and punished: ‘I just think why … God would make me to be like this … because there’s not many things I can do. He made me rather to be different’ (Participant 6, Female, age 18). For Participant 5, her relationship with God helped her to cope with her illness and transplant: ‘I prayed and believed God would make me well …. I myself cannot believe that I recovered so quickly’ (Participant 5, Female, age 20).

## Discussion

The themes highlight the participants’ struggles with transition from paediatric to adult services. The uncertainty, change, loss, and ambivalence illustrate the individualised struggles of coping with a life-threatening illness and an organ transplant at a time of developmental change. Participants described feeling ‘pushed out’ from paediatric services, which left them feeling unwanted and floundering in the unfamiliar surroundings of the adult facility (Crawford et al. 2020). These life experiences affected each adolescent differently, depending on their individual vulnerability and cognitive and behavioural adaptability.

The establishment of a secure base is one of the most important milestones of early childhood development. A secure base facilitates exploration, relationship forming, and is important for safety, stress regulation, adaptability, and development of resilience (Colonnesi et al. 2011; Cortina & Marrone 2003). A child with chronic illness experiences multiple separations, hospitalisations, and traumatic treatment interventions during life (Ødegård 2005). An insecure or disrupted attachment that coincides with mother–child separation because of hospitalisation has been associated with ‘poor social competence in adolescence’ (Calam 2001:3). The disruption of moving from familiar environments to unknown situations is often repeated during normal adolescence, and especially so, for the child with a chronic illness, during the transition of care.

Transition and change are fundamental aspects of every person’s life and can be successfully negotiated with support (Nurser et al. 2018). Adolescence is a period of personal transition, adjustment, and loss (Shifflet-Chila et al. 2016), with the main tasks being identity formation and individuation from family. For adolescents with a transplant, this process is complex; as parents and familiar children’s hospital personnel are replaced by relative strangers in the transition to the adult care facility, compounded by the realisation that as a transplant recipient, true independence may never be possible. The consistency provided at the adult hospital by familiar clinicians, support groups and the presence of successfully transitioned adolescents at the adolescent clinic, eased the transition experience for the participants (Chhiba et al. 2022; Jose et al. 2021).

The challenges for effective management of chronic illness in adolescence are well documented (Crawford et al. 2020; Francis et al. 2018). A successful organ transplant is a life-changing event and comes with a new set of challenges for recipients, their caregivers, and health professionals. There is a perpetual fragile balance between physical, emotional, and psychosocial needs and the management of the transplant, medication, activities, and relationships (Davidson et al. 2017; Tang et al. 2021).

Clinicians in the caring professions have little, if any, training in the management of the specific developmental needs of children entering and experiencing the physical and emotional turmoil of adolescence in the hospital setting (Wright et al. 2017), which may complicate the transition process. Transition services could be improved if clinicians consider and adapt to the needs of the young people and involve them in the transition process, thus maximising their adherence and adaptation (Lozano & Houtrow 2018). Interventions that address attendance, adherence, and family conflict may improve the long-term quality of life in adolescents (Fernandez & Foster 2021; Killian et al. 2018; Richards et al. 2018).

### Strengths and limitations

The findings of this study are limited to the lived experience of adolescent transplant recipients in a public hospital setting and may not reflect the experience of other organ transplant recipients, although they may have similar experiences.

## Conclusions

This study has offered an interpretive description of the lived experience of adolescents who have transitioned from paediatric to adult healthcare services in a South African setting. The findings suggest that consistency and support are key factors in maintaining compliance of the adolescent transplant patient during transition to adult care. All the participants of this study strongly endorsed the need for multiple levels of support, including pre-transition workshops and post-transition support groups with peers who share similar experiences. The implementation of a facilitated psycho-supportive intervention could minimise the risks of mortality and morbidity during the adolescent transition between paediatric and adult services. Carefully planned collaborative preparation and implementation of transition can facilitate adolescent cooperation and adherence, minimise risk and provide the adolescent with the support needed to adapt to living with a transplant as an adult.
